# The Association Between Childhood Maltreatment and Post-Traumatic Stress Disorder (PTSD) Among Young Adults in Northern Syria

**DOI:** 10.1007/s40653-025-00701-5

**Published:** 2025-03-18

**Authors:** Amani Safwat ElBarazi

**Affiliations:** College of Education and Arts, Lusail University, P.O.BOX 9717, Lusail, Doha Qatar

**Keywords:** Childhood maltreatment, Post-traumatic stress disorder (PTSD), Northern Syria

## Abstract

The impact of childhood maltreatment (CM) on post-traumatic stress disorder (PTSD) is a unique and critical context in Northern Syria, a region that has been impacted by protracted conflict and humanitarian crises. Children in this region are at a higher risk of developing both CM and PTSD due to the pervasive displacement, exposure to violence, and socioeconomic instability. These associations are examined in this research, which illuminates the psychological repercussions of adversity in conflict-affected populations. (1) Investigate the prevalence of CM types among young adults exposed to the Syrian conflict; (2) examine the associations between CM exposure and the development of PTSD in young individuals. Syrian people who lives in Northern Syria. Individuals were asked to complete the Childhood Trauma Questionnaire (CTQ) and Posttraumatic Stress Disorder Checklist (PCL-5). A total of 508 people filled out the questionnaire. 55% of the participants suffered from PTSD, also, there was a significant prevalence of childhood abuse among Syrian children (93.7%). From most common to least common, the CM among Syrians was physical neglect (99.4%), emotional neglect (98.8%), emotional abuse (83.1%), physical abuse (34.4%), and sexual abuse (16.1%). The findings from the logistic regression analysis indicated that experiencing physical abuse in childhood notably increased the probability of developing PTSD in adulthood (Odds ratio [OR], 0.7; 95% [CI], 0.6–0.8, *P* <.00). Furthermore, exposure to emotional abuse in childhood significantly increased the probability of developing PTSD in adulthood (Odds ratio [OR], 0.7; 95% [CI], 0.5–0.9, *P* <.01). Childhood exposure to sexual abuse significantly elevates the risk of developing PTSD in adulthood (Odds ratio [OR], 0.7; 95% [CI], 0.6–0.9, *P* <.01). Due to the significant incidence of CM and its robust correlation with PTSD in conflict-affected areas such as Northern Syria, urgent targeted treatments are essential. Treatment strategies should incorporate trauma-focused cognitive behavioral therapy (TF-CBT) and community-based psychosocial support services that are available in humanitarian contexts. Prevention strategies are addressed in the research. One of the study’s **limitations** is that it employs a descriptive cross-sectional design, which does not infer causality. Future research could incorporate longitudinal or experimental designs to provide a more comprehensive understanding of the relationships between variables. Furthermore, the incorporation of qualitative methodologies could provide a more comprehensive understanding of the mechanisms that underlie these associations.

## Introduction

The Syrian conflict, now entering its second decade, has resulted in one of the most significant humanitarian crises of the 21st century. Millions of children have experienced displacement, orphanhood, or extreme violence, profoundly influencing their psychological development (Rizkalla et al., 2020a). In contrast to PTSD resulting from isolated traumatic incidents, Syrian children endure chronic, multifaceted trauma (Hassan et al., 2016), encompassing direct exposure to warfare (including airstrikes, executions, and chemical attacks), enforced displacement, loss of family members, and abuse within precarious refugee environments (Kampalath et al., 2023). PTSD is a significant psychological condition that may develop after experiencing traumatic experiences, characterized by symptoms such as intrusive thoughts, hyperarousal, and emotional numbness (APA, 2013). The experience of PTSD within an Arabic cultural context is significantly shaped by collectivist values, religious coping mechanisms, and the stigma associated with mental health disorders (Al Zomia et al., 2023). Traditional gender norms influence trauma responses, as boys are often socialized to suppress emotions, while girls face an increased risk of sexual violence and forced marriage as strategies for survival in conflict situations (Riley & Vale, 2024; Ford et al., 2015). The influence of religion and faith-based interpretations on the manifestation of symptoms and the approach to treatment is substantial (Rayes et al., 2021). Furthermore, economic collapse resulting from war intensifies PTSD outcomes by elevating instances of child labor, recruitment by armed factions, and exposure to domestic violence, thereby rendering childhood maltreatment inextricable from the overarching trauma of war (Hazer & Gredebäck, 2023). Also, intergenerational transmission of trauma, tough parenting style, control from parents, and parentification mediated and magnified children’s direct and indirect trauma stressors (Veale, 2020).

In Syria, Children have faced arrest, detention, and torture or inhumane conditions in facilities operated by both government and opposition forces. Documentation indicates that children have experienced physical beatings, electrocution, and deprivation of food or medical care (Rizkalla et al., 2024). Extremist groups and warring factions employ public executions as a mechanism of control, subjecting children to severe brutality, such as beheadings and mass killings. Furthermore, numerous pregnant detainees gave birth while incarcerated, and their children resided with them in prison, without the opportunity for the children to leave. In this instance, children were born and raised within prison environments, lacking exposure to the external world and remaining unaware of its existence (Rizkalla et al., 2022). Forced recruitment and child soldiers encompass the enlistment of individuals by armed groups. Multiple factions, including governmental forces, extremist organizations, and rebel militias, have engaged in the forced recruitment of children for roles such as combatants, spies, messengers, and executioners. Certain children are conditioned for combat or utilized as human shields. Brainwashing and Radicalization: Some extremist organizations have indoctrinated children into violent religious extremism, instructing them in combat tactics and idealizing martyrdom.

Hence, in Syria, prolonged armed war, displacement, and instability have fostered an environment where children endure direct trauma, and face heightened risk of CM (Bürgin et al., 2022). Child maltreatment (CM) refers to any form of abuse or neglect a child has endured before turning 18 years old. CM is manifested by physical neglect, emotional neglect, physical abuse, emotional abuse, and sexual abuse (Butchart et al., 2006). The negative effects of CM on healthy development and psychological functioning are well documented (Greeley, 2022; Sege et al., 2017; Cicchetti & Toth, 2005; Springer et al., 2003). Numerous studies have demonstrated that children who were exposed to are more likely to develop mental health disorders such as depression, anxiety, obsessive-compulsive disorder, post-traumatic stress disorder (PTSD), or substance abuse (Oshri et al., 2013; Lippard & Nemeroff, 2020; ElBarazi, 2023). In addition, CM may impede the development of healthy interpersonal connections and present challenges in academic and occupational settings. These challenges may present themselves in diverse forms, including difficulties in establishing trust, diminished self-worth, and emotional regulation (Mehta et al., 2023).

Moreover, maltreatment of children is associated with an increased likelihood of developing physical health problems, the severity of which tends to increase with age (Springer et al., 2003; McEwen & Stellar, 1993; Cicchetti & Handley, 2019; Daigneault et al., 2017; Stein et al., 2013). The long-term health effects of child abuse are negative (Mehta et al., 2023). Individuals with a history of CM are more susceptible to a variety of physical conditions, including cardiovascular diseases, cancer, chronic respiratory diseases (Fergusson et al., 2013), obesity (Dube et al., 2010), poor overall health, and gastrointestinal and cardiorespiratory disorders (Irish et al., 2010).

The Developmental Trauma Theory (DTT) (Cruz et al., 2022), and the Stress-Diathesis Model (Hawn et al., 2022) can be utilized to systematically examine the connection between CM and PTSD in populations affected by conflict. DTT suggests that early-life trauma interferes with neurobiological and emotional development, resulting in enduring psychological effects, including PTSD (Hughes et al., 2017). This framework is especially pertinent in Northern Syria, where extended exposure to violence and instability exacerbates the impacts of CM. The Stress-Diathesis Model posits that individual vulnerabilities, such as genetic predisposition and prior trauma, interact with environmental stressors, including displacement and war exposure, to affect the risk of PTSD. The application of these frameworks facilitates a thorough analysis of trauma-related psychopathology in children affected by conflict and guides the development of targeted intervention strategies.

Applying allostatic load theory could potentially provide insight into how distressing occurrences, such as child maltreatment, can precipitate physical and mental health complications (Sterling & Eyer, 1988). Allostasis involves a lot of different bodily systems working together, like the immune system, the cardiovascular system, cortisol, epinephrine, and anti-inflammatory peptides. Chronic stress and repeated exposure to adversity, such as CM, can lead to long-term physiological and psychological dysregulation. Allostatic load theory suggests that the body’s ability to achieve stability through change, adjusting physiological responses to environmental demands, is affected by chronic stress. The hypothalamic-pituitary-adrenal (HPA) axis plays a central role in allostasis, regulating cortisol secretion. However, chronic stress leads to dysregulated cortisol release, leading to heightened stress responses. Repeated activation of neurobiological stress systems, such as the HPA axis, autonomic nervous system, and immune response, increases vulnerability to psychiatric disorders like PTSD. These neurological consequences include hippocampal atrophy, prefrontal cortex dysfunction, and amygdala hyperactivation. Increased inflammatory markers contribute to mental health disorders and physical illnesses, while dysregulated autonomic nervous system activity exacerbates PTSD symptoms (McLaughlin, 2016). Repeated stress responses can lead to cognitive and emotional impairments, reinforcing maladaptive coping strategies and worsening PTSD outcomes (Brennenstuhl & Fuller-Thomson, 2015; Schiff et al., 2023).

While existing research has established a link between CM and PTSD, limited studies have examined this relationship in the unique context of conflict-affected populations, particularly in Northern Syria. The interplay between CM and PTSD in this setting remains underexplored, especially regarding the differential impact of specific forms of maltreatment (e.g., emotional vs. physical abuse), potential gender differences, and the role of ongoing conflict-related stressors. This study aims to fill this gap by analyzing the distinct contributions of various CM types to PTSD symptom severity. This study aims to investigate the prevalence of CM in Northern Syria and its association with PTSD within the context of ongoing conflict and displacement. Specifically, the study seeks to: (1) determine the prevalence rates of different forms of CM (physical, emotional, and sexual abuse, as well as neglect) among children in Northern Syria; (2) examine the relationship between CM and PTSD symptom severity. Based on existing literature, we hypothesize that (a) higher exposure to CM will be associated with greater PTSD symptom severity, (b) emotional abuse and neglect will have particularly strong associations with PTSD, and (c) displaced children will exhibit heightened vulnerability to both CM and PTSD due to compounded stressors. By addressing these questions, this study aims to provide critical insights for the development of targeted mental health interventions in conflict-affected populations.

The investigation of childhood maltreatment and PTSD in Northern Syria faces with numerous obstacles, such as the inability to obtain dependable data as a result of ongoing conflict and displacement, the potential underreporting of maltreatment due to stigma or fear, and the intricacies of distinguishing between trauma and maltreatment, as well as war-related stressors. Furthermore, the manner in which symptoms are reported or communicated may be influenced by cultural factors, which presents a challenge for standardized assessment tools.

## Methods

### Study Design

This study is a descriptive cross-sectional study.

### Time and Place of the Study

The study was conducted between September 2023 and October 2023. The two-month duration of the study may restrict the thoroughness of data gathering and analysis. To increase participant diversity, recruiting activities were ramped up around peak engagement periods, such as the Syrian Association for Mental Health’s annual conference. Furthermore, snowball sampling was promoted, allowing participants to refer others, therefore increasing outreach among refugee and displaced communities.

### Population and Sample

The Syrian civil war descended into unrest in 2011. Hence, to verify whether the participant experienced the Syrian conflict during their childhood, their age in this research mustn’t surpass 28 years. Child maltreatment, colloquially referred to as CM, comprises a wide range of abuse and neglect that individuals endure before attaining the age of 18. All participants were Syrians living in northern Syria. This study utilized a convenience sampling method, recruiting participants through an online platform affiliated with the Syrian Association for Mental Health (SAMH). Convenience sampling was chosen due to the logistical constraints of conducting research in an active conflict zone, where traditional random sampling is often unfeasible. Inclusion Criteria: (1) being a Syrian individual; (2) being an adult between 18 and 28 years of age. The exclusion criteria were (1) cognitive difficulties and (2) schizophrenia (or any other psychotic disorders).

### Data Collection Tools

Standardized questionnaires and various questions were employed. The study data were collected using:

#### Personal Information

Inquiries consist of sociodemographic, personal, family-related, social, financial, and academic questions. In addition, individuals were questioned about living alone or with a family, physical illness, marital status, and the number of children.

#### Posttraumatic Stress Disorder Checklist (PCL-5)

The PCL-V consists of 20 self-report items (Weathers et al., 2013). On a five-point scale, “0” corresponds to “not at all” and “4” corresponds to “extremely.” Initial research indicates that a PCL-5 score of 33 indicates the presence of PTSD (Blevins et al., 2015). A provisional PTSD diagnosis can be made by treating each item rated 2 = “Moderately” or higher as an endorsed symptom and then applying the DSM-5 diagnostic rule, which requires at least: 1 B item (questions 1–5), 1 C item (questions 6–7), 2 D items (questions 8–14), and 2 E items (questions 15–20). The current study utilized a PCL-5 cutoff score of 33 to indicate the presence of probable PTSD. In the present study, PCl-5 demonstrated strong interrater reliability (α = 0.87). Arabic samples were utilized to validate the Arabic variant of the PCL-V (Ibrahim et al., 2018).

#### The Childhood Trauma Questionnaire-Short Form (CTQ)

Is a self-reported questionnaire administered to adolescents and adults to investigate childhood trauma. As designed by Bernstein and coworkers (Bernstein et al., 1994; Bernstein & Fink, 1998), it is a five-point Likert-type self-report scale. The scale contains five subscales: emotional neglect, physical neglect, physical abuse, emotional abuse, and sexual abuse. It was suggested that for sexual and physical abuse > 5 points, physical neglect, and emotional abuse > 7 points, emotional neglect > 12 points, and as the cut-off score of the total score > 35 points. For the current study, one-week test-retest stability was high (0.89). Arabic samples were utilized to validate the Arabic version of the Childhood Trauma Questionnaire. To avoid cultural sensitivity, Al-Zahrani’s study condensed the five sexual abuse-related questions in the CTQ to a single question. The questionnaire’s Arabic translation has been standardized to the Saudi Arabian dialect (Al-Zahrani, 2005).

### Operational Definitions of Childhood Maltreatment Variables

To maintain clarity and consistency in measurement, the subsequent definitions are applied for each type of CM analyzed in this study:

***Physical abuse*** within the Syrian context encompasses Corporal punishment, such as slapping and beating, is frequently employed in domestic settings and certain schools as a form of discipline. Conflict-related violence, such as the forced recruitment of children by armed groups, domestic stress-induced physical harm, and war-related punishments, may overlap with conventional forms of abuse. Also, restricted healthcare access in conflict zones results in untreated physical injuries (Temple et al., 2018).

***Emotional abuse*** encompasses verbal or non-verbal actions that negatively impact a child’s self-esteem or emotional health. This encompasses threats, humiliation, rejection, excessive criticism, or exposure to domestic violence. Emotional abuse in the Syrian context includes war and displacement can induce parental and societal stress, resulting in increased instances of verbal aggression, public shaming, and threats. Also, children can experience significant psychological stress as a result of witnessing bombings, executions, or the harm or death of family members (Perkins et al., 2018).

***Physical neglect*** refers to the inability to meet a child’s fundamental physical requirements, such as sufficient food, shelter, clothing, hygiene, or medical care, despite the availability of resources. Physical neglect in Syria includes War-related poverty, migration, and infrastructure destruction cause carers to neglect children. Economic hardship has increased child labor, pushing children to work in dangerous conditions without sufficient nourishment or healthcare. Many refugee and conflict zone children lack homes, safe water, and medical care (Habib et al., 2021).

***Emotional neglect*** refers to the inadequate provision of essential emotional support, security, or affection. This includes a consistent lack of attention, neglecting the child’s emotional needs, or failing to provide comfort during distressing circumstances. Within the Syrian context, emotional neglect includes that many carers emotionally unavailable because of the combat, resulting in unintended emotional neglect. War orphans and displaced children may lack emotional support. Besides, due to trauma, many parents may not recognize or respond to their children’s emotional needs (Rizkalla et al., 2020a).

***Sexual abuse*** refers to any instance of forced, coerced, or exploitative sexual contact or exposure involving a child. This encompasses inappropriate touching, penetration, exposure to sexual material, or coercion into sexual acts by an adult or older individual. Girls and boys in Syrian displacement camps, conflict zones, and refugee camps are at danger of sexual exploitation, forced marriage, and trafficking. Early and coerced child marriage has grown as a survival tactic, especially among Syrian refugees.

All variables will be assessed using standardized, culturally adapted tools and self-reports when possible, with modifications implemented to address the unique challenges of data collection in conflict-affected environments.

### Study Process

The participants were recruited using the website of the Syrian Association for Mental Health. An advertisement was posted on the Association’s website to invite people to participate in the research. Participants were invited to complete an online survey, which was distributed via SAMH social media channels, forums, and email lists. Therefore, we kindly request those who have consented to participate in the study to complete the evaluations via online platforms such as Google Forms. On average, each participant required roughly 25–30 min to complete the questions.

### Ethics and Human Subjects Issues

Approval from the British University in Egypt Institutional Review Board was obtained to conduct the study (IRB Protocol CL-2318). Participants signed up for the information sheet and consent form. Participants received a detailed consent form outlining the study’s purpose, potential risks, and their right to withdraw at any time without consequences. The form emphasized that participation was entirely voluntary, and responses were anonymous and confidential, reducing concerns about stigma or repercussions. The study includes trigger warnings & sensitive question sequencing. So, before answering questions related to trauma and PTSD, participants were warned about potentially distressing content and given the option to skip or stop at any time. At the end of the survey, participants were provided with a list of mental health support organizations operating in Syria and neighboring refugee-hosting countries, including: (1) Syrian Association for Mental Health (SAMH) (offering online counseling and mental health resources), (2) International NGOs such as Médecins Sans Frontières (MSF) and the International Committee of the Red Cross (ICRC) providing psychological services in Syria and refugee camps, (3) Self-Help and Coping Strategies: Participants were given access to psychoeducational materials on trauma coping mechanisms, stress management, and emotional regulation techniques.

While direct follow-up was limited due to anonymity, participants were encouraged to seek support if they experienced distress. The study adhered to the Declaration of Helsinki and WHO guidelines on conducting research on trauma-affected populations.

### Outcome Measures

The primary outcome measures were PCL-5 and CTQ. SPSS Sample Power was employed, and statistical analysis ensured that the sample size was sufficient to detect meaningful differences in primary outcomes. A two-tailed significance test with the desired power of 0.80, and a margin of error equal to 5%, were the parameters we chose based on previous research. There is 80% power to identify differences in primary outcomes with (500 participants).

### Data Analysis

The data were evaluated using the IBM SPSS Statistics, version 23 software program. Descriptive statistics (means, standard deviations, frequencies, percentages) were used to describe the sociodemographic and baseline characteristics of this sample. Logistic regressions were utilized to construct odds ratios (ORs) with 95% confidence intervals (95% CIs), independently, to determine whether there is a connection between CM and the likelihood of developing PTSD.

## Results

### Sociodemographic Characteristics

The number of participants who completed the measures was (*N* = 508), 66.3% females and 33.7% males. The mean age of the participants was (*M* = 22.6 ± 3.8) years (min.=18, max.=28). The sociodemographic features of the sample are shown in Table 1. Regarding variables such as marital status, income, place of residence, number of children, family living arrangement, current employment status, and sex, no statistically significant distinctions were identified between individuals who experienced childhood maltreatment and those who did not. However, there was a statistically significant association between CM and adulthood medical illness (odds ratio [*OR*], 0.97; 95% confidence interval [*CI*], 0.96-0.99). Also, there were no statistically significant differences between the sociodemographic traits of people with PTSD and those who did not have it. However, there was a statistically significant association between PTSD and current employment status.

**Table 1:** *Sociodemographic characteristics of the Syrian sample living in Northern Syria.*

Variables		*N* = 508	*%*	*CM*		*PTSD*	
				*χ2*	*Sig*	*χ2*	*Sig*
Sex	Female	337	66.3	1.5	0.5	0.5	0.5
	Male	171	33.7				
Marital Status	Single	283	55.7	2.3	0.4	4.7	0.1
	Married	206	40.6				
	Widow	10	2				
	Divorced	9	1.8				
Education	Primary education	2	0.4	0.1	0.9	0.7	0.9
	Secondary education	4	0.8				
	Higher education	8	1.6				
	University	477	93.9				
	Postgraduate (MsC- Ph.D)	17	3.3				
Place of residence	Aleppo	34	6.7	0.6	0.9	2.5	0.7
	Northern Aleppo Countryside	423	83.3				
	Idlib	26	5.1				
	Homs	16	3.1				
	Damascus	6	1.2				
	Northern Latakia Countryside	3	0.6				
Number of children	0	351	69.1	0.5	0.9	0.4	0.9
	1–2	117	23				
	3–4	40	7.8				
Living with family or friends	No	43	8.5	2.4	0.2	0.05	0.8
	Yes	465	91.5				
Any medical illness	No	178	35	4.6	0.03	1.4	0.2
	Yes	330	65				
Current Employment status	Full time job	202	39.8	3.1	0.3	9.1	0.02
	Part time job	38	7.5				
	Unemployed	15	3				
	Student	253	49.8				
Income	lower than life’s expenses	222	43.7	3.2	0.1	0.1	0.9
	equal to life’s expenses	243	47.8				
	higher than life’s expenses	43	8.5				
Severity of Trauma	No	43	8.5	1.1	0.7	464.6	0.00
	Moderately	85	16.7				
	Mild	112	22				
	Extremely	268	52.8				
Time of trauma	No	43	8.5	0.5	0.9	75.5	0.00
	less than 6 months	155	30.5				
	6–12 months	111	21.9				
	1–5 years	174	34.3				
	5–10 years	25	4.9				

Note. *N* = 508. Participants were on average 22.6 years old (*SD* = 3.8)

### The Prevalence of CM among People in Northern Syria

During the war, there was a significant prevalence of childhood abuse among Syrian children (93.7%). As shown in Table 2, from most common to least common, the CM among Syrians was physical neglect (99.4%), emotional neglect (98.8%), emotional abuse (83.1%), physical abuse (34.4%), and sexual abuse (16.1%).

**Table 2:** The prevalence of CM among people in Northern Syria

Types of CM	No *n*(%)	Yes *n*(%)
Physical Abuse	333 (65.6)	175 (34.4)
Physical Neglect	3 (0.6)	505 (99.4)
Emotional Abuse	86 (16.9)	422 (83.1)
Emotional Neglect	6 (1.2)	502 (98.8)
Sexual Abuse	426 (83.9)	82 (16.1)
Childhood Maltreatment	3 (0.6)	505 (99.4)

### The Association Between Childhood Maltreatment and Post Traumatic Stress Disorder PTSD

According to the results obtained from the Posttraumatic Stress Disorder Checklist (PCL), it was found that a significant proportion of individuals, namely 55.1%, had symptoms indicative of posttraumatic stress disorder (PTSD).

The correlation between CM and adulthood PTSD among individuals residing in Northern Syria is presented in Table 3. The results of logistic regression demonstrated that exposure to physical abuse during childhood significantly increased the likelihood of PTSD in adulthood (Odd ratio [OR], 0.7; 95% confidence interval [CI], 0.6–0.8, *P* <.00). Also, exposure to emotional abuse during childhood significantly increased the likelihood of PTSD in adulthood (Odd ratio [OR], 0.7; 95% confidence interval [CI], 0.5–0.9, *P* <.01). Exposure to sexual abuse during childhood significantly increased the likelihood of PTSD in adulthood (Odd ratio [OR], 0.7; 95% confidence interval [CI], 0.6–0.9, *P* <.01).

**Table 3:** The correlation between of CM and PTSD among people in Northern Syria

Types of CM	*n*(%)	OR	95% CI		*p*
Physical Abuse	175 (34.4)	0.7	0.6	0.8	0.00
Physical Neglect	505 (99.4)	0.6	0.1	2.9	0.59
Emotional Abuse	422 (83.1)	0.7	0.5	0.9	0.01
Emotional Neglect	502 (98.8)	0.3	0.05	1.79	0.09
Sexual Abuse	82 (16.1)	0.7	0.6	0.9	0.01
Childhood Maltreatment	505 (99.4)	0.6	0.1	2.9	0.59

As seen in Table 4, individuals who experienced maltreatment as children have higher PCL scores in adulthood than those who did not endure CM, including intrusive memories, flashbacks, hypervigilance, and avoidance.

**Table 4:** The association between types of CM and the post traumatic stress disorder (PTSD) symptoms

	Physical Abuse		Physical Neglect		Emotional Abuse		Emotional Neglect		Sexual Abuse		Total CM	
	No M (SD)	Yes M (SD)	No M (SD)	Yes M (SD)	No M (SD)	Yes M (SD)	No M (SD)	Yes M (SD)	No M (SD)	Yes M (SD)	No M (SD)	Yes M (SD)
Re-experiencing	9.2(5.9)	10.9(5.8)	11(7.5)	9.8(5.9)	9.3(6.4)	9.9(5.8)	6.6(6.9)	9.8(5.9)	9.6(5.9)	11.1(5.8)	11(7.5)	9.8(5.9)
Avoidance	3.9(2.7)	4.7(2.9)	2.3(1.5)	4.2(2.8)	3.8(2.7)	4.2(2.8)	4(2.4)	4.2(2.8)	4.1(2.8)	4.8(2.8)	2.3(1.5)	4.2(2.8)
Cognitions & Mood	11.7(8.2)	15.8(8.4)	12(7.5)	12.9(8.4)	10.7(8.1)	13.4(8.4)	8.8(6.2)	13(8.4)	12.3(8.3)	16.1(8.3)	12(7.5)	12.9(8.4)
Arousal and reactivity symptoms	9.2(6.6)	12.1(7.6)	9(4.5)	10.2(7.1)	8.6(6)	10.5(7.3)	6.5(5.5)	10.2(7.1)	9.6(6.9)	13.1(7.4)	9(4.5)	10.2(7.1)
Total PTSD	34.2(20.6)	42.9(22.3)	34.3(17.2)	37.2(21.6)	32.5(20.1)	38.2(21.8)	26(15.3)	37.3(21.6)	35.7(21.2)	45.1(22.1)	34.3(17.2)	37.2(21.6)

## Discussion

We aimed this study to investigate the prevalence of CM types among young adults exposed to the Syrian conflict and to examine the associations between CM exposure and the development of (PTSD) in adulthood in young individuals. The findings indicated that among young adult Syrians, the prevalence of CM was very high. The prevalence of childhood maltreatment (CM) among Syrian young adults was arranged in the following order: physical neglect, emotional neglect, physical abuse, emotional abuse, and sexual abuse.

Northern Syria’s socio-political and cultural factors contribute to CM and PTSD. Prolonged conflict, economic collapse, cultural norms, and limited mental health infrastructure contribute to the issue. Political instability, lawlessness, and domestic violence have led to increased exposure to child exploitation and neglect. Economic collapse has led to child labor, forced marriages, and exploitative practices, exacerbating emotional and physical neglect. Traditional Syrian society stigmatizes mental health and trauma, and collectivist values emphasize resilience. Lack of mental health infrastructure and religious practices may not always address the psychological underpinnings of trauma (Beni Yonis et al., 2020; Çeri et al., 2018; Eruyar et al., 2018; Karam et al., 2019).

One possible explanation for our findings of high rates of physical and emotional neglect among Syrian children is that the war has created a chaotic and unstable environment, leading to a breakdown in family structures and support systems. The disturbance might have compelled guardians to place survival ahead of their children’s emotional and physical welfare, leading to the manifestation of neglectful conduct. Furthermore, carers might have become desensitized to the violence and trauma they witnessed daily throughout the conflict, rendering it more difficult for them to identify and effectively address the needs of their children. In addition, during the conflict, there is a lack of food, water, electricity, medical care, and other basic physiological demands. The deprivation of education and fundamental necessities throughout the conflict exacerbated their overall deterioration and future opportunities.

According to our findings, and consistent with previous research (Hager & Runtz, 2012), exposure to maltreatment during childhood is significantly associated with the development of medical conditions in adulthood. Also, past studies showed that exposure to childhood maltreatment has had a profound impact on young adults’ mental and emotional well-being, often leading to long-term psychological consequences such as anxiety, depression, and post-traumatic stress disorder (PTSD) (Kanan & Leão, 2022; Tabur et al., 2019). Consistent with the findings of a prior study (Kakaje et al., 2022), our research revealed that nearly 55% of the participants are afflicted with PTSD. The only significant link was found between physical, emotional, and sexual abuse and the development of PTSD in adults.

While childhood maltreatment is generally associated with PTSD, some forms—such as physical and emotional neglect—may not show a statistically significant relationship in this study. Certain psychosocial and resilience factors may mediate or buffer the impact of neglect on PTSD development such as social support. Unlike overt abuse (e.g., physical or sexual abuse), which involves direct harm, neglect is characterized by the absence of care or emotional responsiveness (Ungar, 2013). In collectivist cultures like Syria’s, extended family networks, religious communities, or peer relationships may compensate for neglect, reducing its long-term psychological impact (Alfadhli, 2018). Research suggests that neglect is more strongly linked to dissociation and emotional dysregulation rather than classical PTSD symptoms like hypervigilance and flashbacks (e.g., Powers et al., 2015). Individuals exposed to neglect may develop avoidant coping mechanisms, making PTSD less apparent compared to those exposed to physical or sexual abuse (Walsh et al., 2010). Also, it could be that in Arabic culture, emotional neglect may be normalized or not perceived as a form of maltreatment, leading to underreporting or different interpretations of its impact. These results can be interpreted using the attachment Theory and Developmental Trauma. Emotional neglect is strongly linked to attachment disruptions, which often result in borderline personality traits, low self-worth, and difficulties in emotional regulation rather than PTSD per se (El Zouki et al., 2024). Some individuals exposed to neglect might develop internalizing disorders (e.g., depression, anxiety) rather than classic PTSD symptoms (Lippard & Nemeroff, 2020).

The significant relationship between CM and PTSD in adulthood can be understood through several psychological and sociological frameworks. These theories explain how early traumatic experiences shape long-term neurobiological, emotional, and social vulnerabilities, increasing the likelihood of developing PTSD later in life. For example, family systems theory posits that physical and emotional ailments of family members may be transmitted unconsciously from one generation to the next (Bowen, 1993). Children of Syrian adults who have experienced war trauma may be impacted by the distressing physical and mental health consequences of war, such as PTSD and somatic symptoms (Rizkalla et al., 2020b). An additional source of stress for the children may be elucidated through the lens of family stress theory, which posits that parenting style and family dynamics are influenced by financial difficulties and everyday challenges. Stress compromises the integrity of familial relationships by interfering with typical family operations (Hill, 1958; Fitzgerald et al., 2020). The adverse economic consequences of war present another considerable challenge for households, impeding parents’ ability to provide their children with optimal care. As a result, children are subjected to cases of childhood maltreatment as a consequence of the implementation of severe parenting practices (Miller & Rasmussen, 2010; Euser et al., 2011; Sim et al., 2018).

CM and PTSD can be explained by Allostatic Load Theory (McEwen & Stellar, 1993). Allostatic overload—chronic hyperarousal or burnout—occurs when childhood abuse overwhelms the body’s stress response regulation. Repeated childhood stress dysregulates the hypothalamic-pituitary-adrenal (HPA) axis, rendering adults more sensitive to trauma. Maltreated people have increased cortisol dysregulation, which increases PTSD risk with adult stresses (McEwen, 2000). In the Syrian context, extended exposure to conflict, displacement, and childhood abuse exacerbates allostatic load, intensifying PTSD severity (Kanan & Leão, 2024). Attachment Theory (Bowlby, 1969; Ainsworth, 1978) helps elucidate the correlation between childhood maltreatment and PTSD. Childhood abuse undermines attachment stability, resulting in insecure or disorganized attachment patterns that increase susceptibility to PTSD in adulthood. Syrian children exposed to war and experiencing disturbed attachment owing to parental loss or displacement may develop anxious-preoccupied attachment, increasing their vulnerability to PTSD in adulthood. An alternative elucidation of the Dual Representation Theory of PTSD (Brewin et al., 1996). PTSD arises when stress is recorded in two conflicting memory systems: (1) Situationally Accessible Memory (SAM): Sensory-driven, involuntary traumatic memories that elicit flashbacks. (2) Verbally Accessible Memory (VAM): Contextualized, aware remembrance of trauma. Syrian children who experienced bombings, torture, or familial fatalities may acquire PTSD in adulthood when dissociated traumatic memories resurface during periods of stress. Betrayal Trauma Theory (Freyd, 1996; Freyd & Birrell, 2013) elucidates the foundations. When abuse is inflicted by a trusted carer, the brain inhibits painful memories as a survival strategy. A child who experienced physical abuse by a family member during wartime may repress the memories but subsequently exhibit PTSD symptoms when confronted with reminders of war or familial discord. Social Learning Theory (Bandura, 1977, 1986) posits that individuals acquire behaviors through observation and imitation, indicating that childhood abuse imparts maladaptive coping mechanisms. Within the context of Syria, A child raised in a warzone where violence is commonplace may cultivate aggressive coping strategies or emotional detachment, increasing the likelihood of PTSD when confront with stress in later life. The Stress Process Model (Pearlin et al., 1981) posits that the cumulative impact of stresses throughout one’s life heightens the probability of developing PTSD. A Syrian individual who experienced childhood abuse may encounter challenges such as unemployment, housing instability, and prejudice, exacerbating their PTSD symptoms.

Given the high prevalence of CM and its strong association with PTSD in conflict-affected regions like Northern Syria, targeted interventions are urgently needed. Treatment approaches should include trauma-focused cognitive behavioral therapy (TF-CBT) and community-based psychosocial support programs that are accessible in humanitarian settings (Alzaghoul et al., 2022). Preventive measures should prioritize strengthening child protection systems, training caregivers and educators to recognize and respond to CM and integrating mental health services into primary healthcare and school-based programs. Implementation in a conflict setting requires collaboration with local organizations, mobile mental health units, and digital platforms to reach displaced and at-risk children. Future research should assess the feasibility and effectiveness of such interventions in this challenging context.

The essential find emphasizes the significant and long-lasting consequences of CM on mental health results, specifically within the context of a conflict-ravaged area such as Syria. The correlation between CM and PTSD indicates the necessity for focused interventions and support networks to tackle the enduring psychological consequences of CM. These initiatives may present viable approaches to enhance the mental health of those impacted. In addition to adding to the expanding body of knowledge on CM, these findings emphasize the critical nature of developing all-encompassing approaches to combat and prevent child maltreatment in regions afflicted by conflict, such as Syria. Policy recommendations advocate for mental health interventions to focus on family units rather than only on individuals. Community-based support networks may mitigate the effects of childhood abuse on PTSD. Programs should prioritize the reconstruction of familial roles and structures, taking into account the cultural importance of family honor, collectivism, and resilience narratives within Syrian communities.

### Limitations and Future Directions

First, the reliance of this research on self-report measures presents a notable constraint concerning the willingness and ability of the participants to divulge private information concerning childhood maltreatment (CM) and post-traumatic stress disorder (PTSD). Second, it is crucial to specify that a cross-sectional methodology was employed to collect data. The results of the research demonstrated the existence of a correlation among the variables being examined. It is essential to note, nevertheless, that the lack of experimental manipulation in the research design precludes any inference of causality from this correlation. Further inquiries may utilize a larger sample size to examine the potential correlation between PTSD and CM. Third, a two-month timeframe may limit the overall number of participants, hence diminishing statistical power and generalizability. Consequently, recognizing that longitudinal research (e.g., a follow-up survey at 6 or 12 months) might elucidate PTSD development and corroborate early findings. Additionally, using longitudinal methodologies in forthcoming study to evaluate temporal changes, especially for displaced populations enduring ongoing trauma exposure. Fourth, recruitment through an online platform might introduce selection bias, as that convenience sample is non-random, limiting generalization to the full Syrian population. Internet connectivity, digital literacy, and mental health service knowledge are required for participation, which may exclude rural, refugee, and low-income people. Also, SAMH participants may be more interested in mental health concerns, over-representing those seeking psychological treatment. The study recognizes that due to sampling limitations, the findings may not adequately reflect the wider Syrian population, especially among displaced individuals and those with restricted access to mental health resources. Future research may improve representativeness through: (1) Employing mixed-method sampling, such as integrating offline recruitment in refugee camps, clinics, or community centers. (2) Analyzing sample characteristics against established Syrian demographic distributions (e.g., gender, age, socioeconomic status) to evaluate potential biases. (3) Implementing follow-up studies with focused outreach to marginalized populations, including internally displaced people (IDPs) and rural communities.

## Conclusion

The prevalence of physical and emotional neglect among Syrian children was substantially heightened due to the prevailing circumstances of the conflict. A strong association can be observed between adulthood and the development of post-traumatic stress disorder (PTSD), which is characterized by physical, emotional, and sexual abuse. The correlation mentioned above highlights the long-lasting effects of abuse on the mental health of Syrian children, as it may progress to (PTSD) in adulthood. It is critical to prioritize the prevention and resolution of all forms of abuse to ensure the well-being and future opportunities of these susceptible children.

## Data Availability

The corresponding author can provide reasonable access to the datasets created during and/or analyzed during the current investigation.
